# CYP2E1 Sensitizes the Liver to LPS- and TNF **α**-Induced Toxicity via Elevated Oxidative and Nitrosative Stress and Activation of ASK-1 and JNK Mitogen-Activated Kinases

**DOI:** 10.1155/2012/582790

**Published:** 2011-10-18

**Authors:** Arthur I. Cederbaum, Lili Yang, Xiaodong Wang, Defeng Wu

**Affiliations:** Department of Pharmacology and Systems Therapeutics, Mount Sinai School of Medicine, P.O. Box 1603, One Gustave L. Levy Place, New York, NY 10029, USA

## Abstract

The mechanisms by which alcohol causes cell injury are not clear. A major mechanism is the role of lipid peroxidation and oxidative stress in alcohol toxicity. Many pathways have been suggested to play a role in how alcohol induces oxidative stress. Considerable attention has been given to alcohol elevated production of lipopolysaccharide (LPS) and TNF**α** and to alcohol induction of CYP2E1. These two pathways are not exclusive of each other; however, interactions between them, have not been extensively evaluated. Increased oxidative stress from induction of CYP2E1 sensitizes hepatocytes to LPS and TNF**α** toxicity and oxidants, activation of inducible nitric oxide synthase and p38 and JNK MAP kinases, and mitochondrial dysfunction are downstream mediators of this CYP2E1-LPS/TNF**α**-potentiated hepatotoxicity. This paper will summarize studies showing potentiated interactions between these two risk factors in promoting liver injury and the mechanisms involved including activation of the mitogen-activated kinase kinase kinase ASK-1. Decreasing either cytosolic or mitochondrial thioredoxin in HepG2 cells expressing CYP2E1 causes loss of cell viability and elevated oxidative stress via an ASK-1/JNK-dependent mechanism. We hypothesize that similar interactions occur as a result of ethanol induction of CYP2E1 and TNF**α**.

## 1. Introduction

The ability of acute and chronic ethanol treatment to increase production of reactive oxygen species and enhance peroxidation of lipids, protein, and DNA has been demonstrated in a variety of systems, cells, and species, including humans [1]. Despite a tremendous growth in understanding alcohol metabolism and actions, the mechanism(s) by which alcohol causes cell injury are still not clear. A variety of leading mechanisms have been briefly summarized [2–4], and it is likely that many of them ultimately converge as they reflect a spectrum of the organism's response to the myriad of direct and indirect actions of alcohol. A major mechanism that is a focus of considerable research is the role of lipid peroxidation and oxidative stress in alcohol toxicity. Under certain conditions, such as acute or chronic alcohol exposure, production of reactive oxygen species (ROS) is enhanced and/or the level or activity of antioxidants is reduced. The resulting state, which is characterized by a disturbance in the balance between ROS production, on one hand, and ROS removal and repair of damaged complex molecules, on the other is called oxidative stress.

 ROS have been implicated in many of the major diseases that plague mankind, including the toxicity of O_2_ itself; hyperbaric O_2_; ischemia-reperfusion injury; cardiovascular diseases; atherosclerosis; carcinogenesis; diabetes; neurodegenerative diseases, including Parkinson's disease and Alzheimer's disease; toxicity of heavy metals, for example, iron; asbestos injury; radiation injury; vitamin deficiency; drug (e.g., redox cycling agents) toxicity; aging; inflammation; smoke toxicity; emphysema; toxicity of acute and chronic ethanol treatment [2–6]. ROS can be produced from many systems in cells including the mitochondrial respiratory chain [7], the cytochrome P450s [8, 9], oxidative enzymes such as xanthine oxidase, aldehyde oxidase, cyclooxygenase, monoamine oxidase, and the NADPH oxidase complex [10], autooxidation of heme proteins such as ferrohemoglobin or myoglobin, or biochemicals such as catecholamines, quinones, or tetrahydrobiopterins. In addition to these cellular sources of ROS, environmental sources of ROS include radiation, UV light, smoke, and certain drugs which are metabolized to radical intermediates or which can redox cycle. ROS are toxic to cells because they can react with most cellular macromolecules inactiving enzymes or denaturing proteins, causing DNA damage such as strand breaks, base removal, or base modifications which can result in mutation, peroxidation of lipids which can result in destruction of biological membranes and produce reactive aldehydic products such as malondialdehyde or 4-hydroxynonenal. A variety of enzymatic and non-enzymatic mechanisms have evolved to protect cells against ROS, including the superoxide dismutases, which remove O_2_^−^; catalase and the glutathione (GSH) peroxidase system which remove H_2_O_2_; glutathione transferases which can remove reactive intermediates and lipid aldehydes, metallothioneins, heme oxygenase, thioredoxin which remove various ROS; ceruloplasmin and ferritin which help remove metals such as iron which promote oxidative reactions; nonenzymatic, low-molecular-weight antioxidants such as GSH itself, vitamin E, ascorbate (vitamin C), vitamin A, ubiquinone, uric acid, and bilirubin [11, 12]. Oxidative stress or toxicity by ROS reflects a balance between the rates of production of ROS compared to the rates of removal of ROS plus repair of damaged cellular macromolecules. While excess ROS can cause toxicity, macrophages and neutrophils contain an NADPH oxidase which produces ROS to destroy foreign organisms [13], and the enzyme myeloperoxidase catalyzes a reaction between H_2_O_2_ and chloride to produce the powerful oxidant hypochlorite (bleach) to help destroy foreign invaders. In addition, ROS at low concentrations, especially H_2_O_2_, may be important in signal transduction mechanisms in cells and thus be involved in cellular physiology and metabolism [14].

 Many pathways have been suggested to play a key role in how ethanol induces “oxidative stress” [1–4]. Some of these include redox state changes (decrease in the NAD^+^/NADH redox ratio) produced as a result of ethanol oxidation by alcohol and aldehyde dehydrogenases; production of the reactive product acetaldehyde as a consequence of ethanol oxidation by all major oxidative pathways; damage to mitochondria which results in decreased ATP production; direct or membrane effects caused by hydrophobic ethanol interaction with either phospholipids or protein components or enzymes; ethanol-induced hypoxia, especially in the pericentral zone of the liver acinus as oxygen is consumed in order for the liver to detoxify ethanol via oxidation; ethanol effects on the immune system and altered cytokine production; ethanol-induced increase in bacterial-derived endotoxin with subsequent activation of Kupffer cells; ethanol induction of CYP2E1; ethanol mobilization of iron which results in enhanced levels of low-molecular-weight nonheme iron; effects on antioxidant enzymes and chemicals, particularly mitochondrial and cytosolic glutathione; one electron oxidation of ethanol to the 1-hydroxy ethyl radical; conversion of xanthine dehydrogenase to the xanthine oxidase form. Again, many of these pathways are not exclusive of one another, and it is likely that several, indeed many, systems contribute to the ability of ethanol to induce a state of oxidative stress.

## 2. Kupffer Cells and Alcoholic Liver Disease

Kupffer cells are stimulated by chronic ethanol treatment to produce free radicals and cytokines, including TNF*α*, which plays a role in ALD [15, 16]. This stimulation is mediated by bacterial-derived endotoxin, and ALD is decreased when gram-negative bacteria are depleted from the gut by treatment with lactobacillus or antibiotics [17]. The TNF*α* receptor superfamily consists of several members sharing a sequence homology, the death domain, located in the intracellular portion of the receptor. These “death” receptors, including Fas, TNF-R1, and TRAIL-R1/TRAIL-R2, are expressed in hepatocytes and when stimulated by their respective ligands, FasL, TNF*α*, or TRAIL, hepatocyte injury can occur [18]. Lipopolysaccharide (LPS) is a component of the outer wall of gram-negative bacteria that normally inhabit the gut. LPS penetrates the gut epithelium only in trace amounts; however, LPS absorption can be elevated under pathophysiological conditions such as alcoholic liver disease [19]. When LPS is released from gram-negative bacteria and enters the blood stream, the liver tightly regulates the entry and processing of LPS by virtue of its ability to clear LPS and respond to LPS [20]. In addition to its ability to clear LPS, the liver also responds to LPS and produces cytokines. LPS directly causes liver injury by mechanisms involving inflammatory cells such as Kupffer cells, and chemical mediators such as superoxide, nitric oxide, and tumor necrosis factor (TNF*α*) and other cytokines [21–23]. In addition, LPS potentiates liver damage induced by hepatotoxins including ethanol [24–29]. In experimental alcoholic liver disease, the combination of LPS and chronic ethanol produce hepatic necrosis and inflammation [27–29]. Ethanol alters gut microflora, the source of LPS, and ethanol increases the permeability of the gut, thus increasing the distribution of LPS from the gut into the portal circulation (endotoxemia). This causes activation of Kupffer cells, the resident macrophages in liver, resulting in release of chemical mediators including cytokines and reactive oxygen species (ROS), and subsequently, alcoholic liver disease [30]. Destruction of Kupffer cells with gadolinium chloride attenuated ALD [15]. A major advance was the finding that anti-TNF*α* antibodies protect against ALD [16]. NADPH oxidase was identified as a key enzyme for generating ROS in Kupffer cells after ethanol treatment [31]. Moreover, in mice deficient in a subunit of NADPH oxidase, p47phox, the ethanol-induced increase in ROS and TNF*α* and liver injury was decreased [32]. The role of TNF*α* in ALD was further validated by the findings that the ethanol-induced pathology was nearly blocked in TNF*α* receptor1 knockout mice [33]. 

The transcription factor nuclear factor-kappaB (NF-*κ*B) in Kupffer cells regulates activation of many inflammatory genes, including TNF*α*. Endotoxin activates NF-*κ*B, leading to the hypothesis that inhibition of NF-*κ*B in Kupffer cells would prevent ALD [34]. Administration of an adenovirus encoding for the IkB superrepressor to rats chronically infused with ethanol blunted the ethanol-induced activation of NF-*κ*B, TNF*α* production, and pathological changes. A general scheme to explain these results is that chronic ethanol treatment elevates endotoxin levels, endotoxin activates Kupffer cells to produce free radicals via NADPH oxidase, the free radicals activate NF-*κ*B, leading to an increase in production of TNF*α*, followed eventually by tissue damage [29].

## 3. CYP2E1

 CYP2E1 metabolizes a variety of small, hydrophobic substrates including solvents such as chloroform and carbon tetrachloride, aromatic hydrocarbons such as benzene and toluene, alcohols such as ethanol and pentanol, aldehydes such as acetaldehyde, halogenated anesthetics such as enflurane and halothane, nitrosamines such as N,N-dimethylnitrosamine, and drugs such as chlorzoxazone and acetaminophen [35–41]. From a toxicological point of view, interest in CYP2E1 revolves around the ability of this P450 to metabolize and activate many toxicologically important compounds such as ethanol, carbon tetrachloride, acetaminophen, benzene, halothane, and many other halogenated substrates. Procarcinogens including nitrosamines and azo compounds are effective substrates for CYP2E1. Toxicity by the above compounds is enhanced after induction of CYP2E1, for example, by ethanol treatment, and toxicity is reduced by inhibitors of CYP2E1 or in CYP2E1 knockout mice [42]. 

 Molecular oxygen itself is likely to be the most important substrate for CYP2E1 [8, 9]. CYP2E1, relative to several other P450 enzymes, displays high NADPH oxidase activity as it appears to be poorly coupled with NADPH-cytochrome P450 reductase [43, 44]. CYP2E1 was the most efficient P450 enzyme in the initiation of NADPH-dependent lipid peroxidation in reconstituted membranes among five different P450 forms investigated. Furthermore, anti-CYP2E1 IgG inhibited microsomal NADPH oxidase activity and microsomal lipid peroxidation dependent on P450, but not lipid peroxidation initiated by the action of NADPH-cytochrome P450 reductase [43]. In our laboratory, we found that microsomes isolated from rats fed ethanol chronically were about twofold to threefold more reactive in generating superoxide radical and H_2_O_2,_ and in the presence of ferric complexes, in generating hydroxyl radical and undergoing lipid peroxidation [45–48]. CYP2E1 levels were elevated about threefold to fivefold in liver microsomes after feeding rats the Lieber-DeCarli diet for four weeks. In all the above systems, the enhanced effectiveness of microsomes isolated from the ethanol-fed rats was prevented by addition of chemical inhibitors of CYP2E1 and by polyclonal antibody raised against CYP2E1 purified from pyrazole-treated rats, confirming that the increased activity in these microsomes was due to CYP2E1. 

 Since CYP2E1 can generate ROS during its catalytic cycle, and its levels are elevated by chronic treatment with ethanol, CYP2E1 has been suggested as a major contributor to ethanol-induced oxidative stress, and to ethanol-induced liver injury [49–53]. Experimentally, a decrease in CYP2E1 induction was found to be associated with a reduction in alcohol-induced liver injury [54–58]. A CYP2E1 transgenic mouse model was developed that overexpressed CYP2E1. When treated with ethanol, the CYP2E1 overexpressing mice displayed higher transaminase levels and histological features of liver injury compared with the control mice [59]. We developed an adenoviral vector which expresses human CYP2E1 and showed that infection of HepG2 cells with this adenovirus potentiated acetaminophen toxicity as compared to HepG2 cells infected with a LacZ expressing adenovirus [60]. Administration of the CYP2E1 adenovirus in vivo to mice elevated CYP2E1 levels and activity and produced significant liver injury compared to the LacZ-infected mice as reflected by histopathology and elevated transaminase levels [61]. However, other studies suggested that CYP2E1 may not play a role in alcohol liver injury based upon studies with gadolinium chloride or CYP2E1 knockout mice [62, 63]. Bradford et al. [64] using CYP2E1 and NADPH oxidase knockout mice concluded that CYP2E1 was required for ethanol induction of oxidative stress to DNA, whereas NADPH oxidase was required for ethanol-induced liver injury. As mentioned earlier, it is likely that several mechanisms contribute to alcohol-induced liver injury and that ethanol-induced oxidative stress is likely to arise from several sources, including CYP2E1, mitochondria, and activated Kupffer cells.

## 4. LPS/TNF***α***-CYP2E1 Interactions

As discussed above, abnormal cytokine metabolism is a major feature of alcoholic liver disease. Rats chronically fed ethanol were more sensitive to the hepatotoxic effects of administration of LPS and had higher plasma levels of TNF*α* than control rats [65, 66]. In the intragastric model of chronic ethanol administration, the development of liver injury coincided with an increase in TNF*α*, associated with an increase in serum LPS [29]. Anti-TNF*α* antibody prevented alcohol liver injury in rats [16], and mice lacking the TNFR1 receptor did not develop alcohol liver injury [33]. Taken as a whole, these and other studies clearly implicate TNF*α* as a major risk factor for the development of alcoholic liver injury. One complication in this central role for TNF*α* is that hepatocytes are normally resistant to TNF*α*-induced toxicity. This led to the hypothesis that besides elevating TNF*α*, alcohol somehow sensitizes or primes the liver to become susceptible to TNF*α* [67, 68]. Known factors which sensitize the liver to TNF*α* are inhibitors of mRNA or protein synthesis, which likely prevent the synthesis of protective factors, inhibition of NF-*κ*B activation in hepatocytes to lower synthesis of such protective factors, depletion of GSH, especially mitochondrial GSH, lowering of S-adenosyl methionine (SAM) coupled to elevation of S-adenosyl homocysteine (SAH), that is, a decline in the SAM/SAH ratio, or inhibition of the proteasome. Combined treatment with ethanol plus TNF*α* is more toxic to hepatocytes and HepG2 E47 cells which express high levels of CYP2E1 than control hepatocytes with lower levels of CYP2E1 or HepG2 C34 cells which do not express CYP2E1 [69]. RALA hepatocytes with increased expression of CYP2E1 were sensitized to TNF*α*-mediated cell death [70]. These results suggest that increased oxidative stress from CYP2E1 may sensitize isolated hepatocytes to TNF*α*-induced toxicity. 

 Either LPS or CYP2E1 is considered independent risk factors involved in alcoholic liver disease, but mutual relationships or interactions between them are unknown. We initiated studies to evaluate whether CYP2E1 contributes or potentiates LPS- or TNF*α*-mediated liver injury in vivo. These studies may provide an experimental model to better understand mechanisms of ethanol-induced liver damage. 

## 5. Pyrazole Potentiates LPS Toxicity [71, 72]

**Figure 1:**
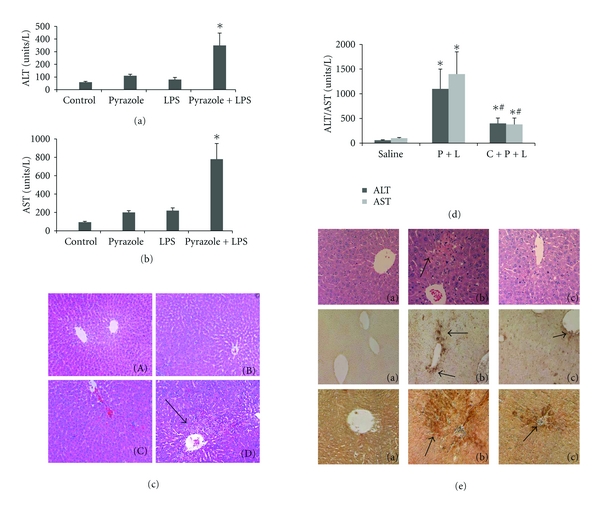
Effect of pyrazole or LPS or LPS plus pyrazole on serum ALT (a) or AST (b) or liver histopathology (c). In (c), panels refer to (A) saline; (B) pyrazole-treated; (C) LPS-treated; (D) LPS plus pyrazole treated. Arrows show necrotic foci with inflammatory cell infiltration. Note: combined treatment with pyrazole plus TNF*α* produces liver injury. The CYP2E1 inhibitor, chlormethiazole (CMZ), protects against LPS plus pyrazole toxicity in mice. (d) ALT/AST levels. Sal: Saline-treated; P + L: pyrazole plus LPS treated: C + P + L, CMZ plus pyrazole plus LPS treated. (e) histopathology (top panels); 3-nitrotyrosine protein adducts (middle panels); 4-hydroxynonenal adducts (bottom panels). In all panels, panel (a) is the saline treated; panel (b) is the LPS plus pyrazole treated; panel (c) refers to the CMZ plus LPS plus pyrazole treated.

 Male, Sprague-Dawley rats (160–180 g) were injected intraperitoneally with pyrazole (PY), 200 mg per kg body wt, once a day for 2 days to induce CYP2E1. After an overnight fast, either saline or LPS (Sigma, serotype 055: BS, 10 mg/kg body wt) was injected via the tail vein. Rats were killed 8–10 hr after the LPS or saline injection and blood and liver tissue collected. Neither pyrazole alone or LPS alone caused liver injury as reflected by transaminase (ALT, AST) levels or liver histopathology (Figures 1(a) and 1(b)). However, the combination of LPS plus pyrazole increased AST and ALT levels about fourfold over the levels in the pyrazole alone or LPS alone groups (Figures 1(a) and 1(b)). LPS-plus-pyrazole-treatment induced extensive necrosis of hepatocytes, mainly located both in periportal and pericentral zones of the liver, accompanied by strong infiltration of inflammatory cells (Figure 1(c)). LPS alone treatment caused some apoptosis and activation of caspases 3 and 9, whereas pyrazole treatment alone had no effect. LPS plus pyrazole treatment was not any more effective than LPS alone in increasing apoptosis, unlike the increases in necrosis and inflammation.

 To assess whether oxidative stress occurs after the various treatments, malondialdehyde (MDA) levels as a reflection of lipid peroxidation were assayed. Whereas pyrazole alone or LPS alone did not elevate MDA levels over those found with saline controls, the combination of LPS plus pyrazole increased MDA levels about 65% (*P* < 0.05 compared to the other 3 groups). Protein carbonyl formation as a marker for oxidized protein formation was determined. Low levels of protein carbonyls were found in saline control livers. Treatment with either LPS alone or pyrazole alone elevated protein carbonyl levels; however, striking increases in protein carbonyls were found in the combined LPS plus pyrazole group. In situ detection of superoxide was measured using the oxidation-dependent fluorescent dye dihydroethidium. Red fluorescence was weak in saline control livers, was slightly increased in either the LPS or pyrazole livers, and was highest in the LPS plus pyrazole livers. 3-Nitrotyrosine (3-NT) protein adducts were detected by a slot blot technique. 3-NT adducts were highest in livers from the LPS-plus-pyrazole-treated mice. Thus, several parameters of oxidative/nitrosative stress were elevated in livers from the LPS plus pyrazole-treated mice.

 CYP2E1 catalytic activity (oxidation of P-nitrophenol to p-nitrocatechol) was increased about 2-fold by either the pyrazole alone or the pyrazole plus LPS treatments. LPS alone slightly but not significantly decreased CYP2E1 activity. Levels of CYP2E1 protein, measured by immunoblot analysis, showed similar trends, being increased about 2-fold by pyrazole or pyrazole plus LPS treatments. These results show that pyrazole treatment enhanced LPS-induced necrosis, not apoptosis. This enhanced liver injury is associated with elevated levels of CYP2E1 and increased oxidative/nitrosative stress generated by the combination of LPS plus elevated CYP2E1.

 To validate the role of CYP2E1 in the potentiation of LPS toxicity by pyrazole, experiments with chlormethiazole (CMZ) an inhibitor of CYP2E1 and with CYP2E1 knockout mice were carried out [71]. C57BL/6 mice were injected intraperitoneally with pyrazole, 150 mg/kg body wt once a day for 2 days or 0.9% saline. After an overnight fast, LPS, 4 mg/kg body wt, or saline was injected IP. CMZ was injected in some mice at a concentration of 50 mg/kg body wt 15 hours before and 30 minutes after the LPS treatment. Mice were killed 3, 8, or 24 h after LPS or saline injection. In other experiments, CYP2E1 knockout mice, kindly provided by Dr. Frank Gonzalez, NCI, NIH, and their genetic background SV129 controls were treated with pyrazole and LPS as above. Initial experiments showed that neither pyrazole alone nor LPS alone produced liver injury under those conditions. However, the LPS-plus-pyrazole-treatment produced significant liver injury in mice, as was previously shown in rats. Little injury occurred at 3 or 8 hr after the LPS administration, but did occur at 24 h. The injury in the LPS-plus-pyrazole-treated mice was associated with an elevation in oxidative/nitrosative stress as reflected by increases in 3-NT and 4-hydroxynonenal (HNE) protein adducts. Administration of CMZ to the LPS-plus-pyrazole-treated mice decreased the elevated ALT and AST levels by about 55 and 65%, respectively, (Figure 1(d)). Pathological evaluation showed large necrotic areas in the livers from the LPS-plus-pyrazole-treated-mice, but only small necrotic foci were observed after treatment with CMZ (Figure 1(e)). The treatment with CMZ also lowered the elevated oxidative/nitrosative stress produced by the LPS plus pyrazole treatment as only weak signals for formation of 4-HNE adducts and 3-NT adducts were found after the CMZ treatment (Figure 1(e)). The pyrazole plus LPS treatment produced a 2-fold increase in CYP2E1 catalytic activity, which was prevented after the administration of CMZ. Thus, CMZ blocked the elevation of CYP2E1 in the LPS-plus-pyrazole-treated mice, and this was associated with a decline in oxidative/nitrosative stress and blunting of liver injury. 

 To further evaluate a role for CYP2E1 in the LPS plus pyrazole toxicity, CYP2E1 knockout or wild-type control SV129 mice were treated with LPS plus pyrazole. As with C57Bl/6 mice, liver injury was observed in the wild-type SV129 mice treated with LPS plus pyrazole, but not mice treated with LPS alone or pyrazole alone. Serum ALT and AST levels were about 50% lower in LPS-plus-pyrazole-treated CYP2E1 knockout mice as compared to wild-type mice. Pathological evaluation showed large necrotic areas and widespread necrotic foci in wild-type mice, whereas almost normal histology was found in the LPS-plus-pyrazole-treated CYP2E1 knockout mice. Positive TUNEL staining was also significantly lower in the CYP2E1 null mice compared to wild-type mice. Immunoblots confirmed the absence of CYP2E1 protein in the knockout mice, while strong signals from CYP2E1 were detected in immunoblots of the wild type mice. Thus, in both rats and mice, the CYP2E1 inducer pyrazole potentiates LPS-induced liver injury. This potentiation is associated with elevated oxidative/nitrosative stress and is blocked by the CYP2E1 inhibitor CMZ and blunted in CYP2E1 knockout mice. We hypothesize that CYP2E1-mediated oxidative stress may synergize with LPS-generated oxidative stress in this model to produce liver injury.

## 6. Pyrazole Potentiates TNF***α*** Toxicity [73, 74]

Since TNF*α* levels are elevated after LPS administration and TNF*α* plays an important role in the effects of LPS, we determined if pyrazole treatment to induce CYP2E1 potentiates TNF*α* toxicity as it did with LPS toxicity. Basically, the same approaches described above were used, with injection of TNF*α* (50 ug/kg body wt.) replacing the LPS treatment.

**Figure 2:**
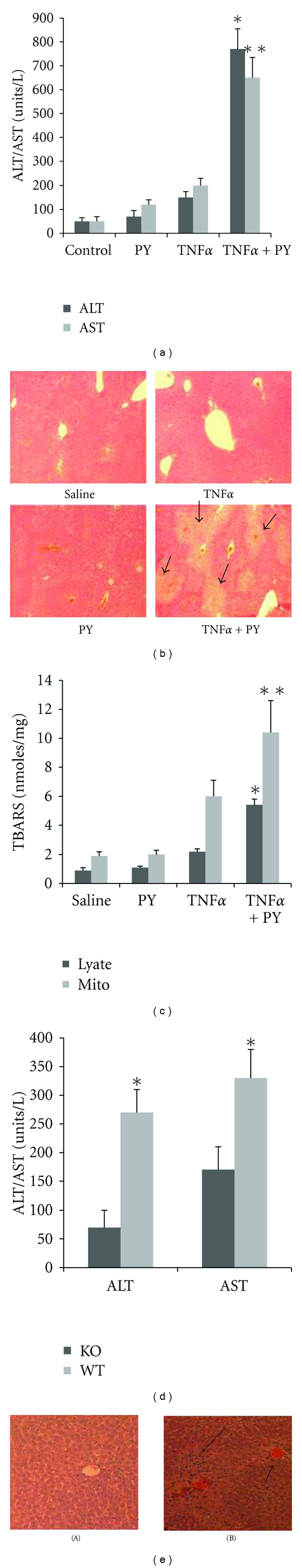
Pyrazole potentiates TNF*α* hepatotoxicity and oxidative stress in mice. Mice were treated with either saline or pyrazole alone or TNF*α* alone or pyrazole plus TNF*α* followed by assays for (a) serum ALT/AST, (b) histopathology (arrows show necrotic zones), (c) lipid peroxidation as reflected by levels of TBARs in liver cell lysates and in isolated mitochondrial fractions. Note: combined treatment with TNF*α* plus pyrazole produces liver injury. (d) Serum ALT and AST levels in pyrazole plus TNF*α*-treated wild type (WT) and CYP2E1 knockout (KO) mice. (e) Histopathology in pyrazole plus TNF*α*-treated KO (panel A) and WT (panel B). Note: liver injury is decreased in CYP2E1 knockout mice compared to WT mice.

Figure 2(a) shows that ALT and AST levels were low in the saline control mice and in the pyrazole-treated mice challenged with saline. Treatment of control mice with TNF*α* elevated transaminase levels by about 2-3-fold. Treatment of the pyrazole mice with TNF*α* elevated transaminase levels more than 3-fold over the TNF*α*-saline control treated mice. Liver sections were stained with H&E for morphological evaluation. The saline and TNF*α* treated mice showed normal liver morphology. Liver from pyrazole treated mice showed some vacuolar degeneration. Liver from the TNF*α*-plus-pyrazole-treated mice showed several necrotic loci (arrows), and typical pathology morphology changes including nuclear pyknosis, karyorrhexis, and karyolysis were observed (Figure 2(b)). The treatment with pyrazole did not significantly alter the levels of thiobarbituric acid-reactive substrates (TBARS) in the total liver extract or the mitochondria (Figure 2(c)). TNF*α* treatment of control mice elevated levels of TBARS about 2-3 fold. TBARS in the homogenates and the mitochondria were further elevated when TNF*α* was administered to the pyrazole-treated mice. Highest liver and mitochondrial TBARs levels were observed in the pyrazole-plus-TNF*α*-treated mice (Figure 2(c)). Liver GSH levels were similar in the saline, pyrazole-treated, and TNF*α*-treated mice but were decreased about 40% in the liver extracts from the pyrazole-plus-TNF*α*-treated mice. GSH levels were lowered 40% in the liver mitochondria from the pyrazole plus TNF*α*-treated mice compared to the TNF*α* alone treated mice. These results suggest that the combined pyrazole plus TNF*α* treatment produces elevated oxidative stress in the liver compared to TNF*α* alone or pyrazole alone, and that mitochondrial oxidative stress may occur in livers of the pyrazole-plus-TNF*α*-treated mice.

 As expected, CYP2E1 activity as reflected by the NADPH-dependent microsomal oxidation of p-nitrophenol and the content of CYP2E1 (Western blot analysis) were elevated 2-to 3-fold by pyrazole or by pyrazole plus TNF*α* treatment, over the saline or TNF*α* alone treated mice. Thus, TNF*α* alone or in combination with pyrazole did not alter CYP2E1 activity or content. Also, induction of CYP2E1 alone by pyrazole is not sufficient to induce liver injury; rather, a second “hit,” for example, TNF*α* is required. What is the evidence that induction of CYP2E1 by pyrazole is important for the elevated injury found in the pyrazole-plus-TNF*α*-treated mice? We used CYP2E1 knockout mice to address this question. Large increases in ALT and AST levels were found after TNF*α* administration to pyrazole-treated SV129 wild type mice. TNF*α* treatment of pyrazole-treated CYP2E1 knockout mice did not elevate transaminase levels (Figure 2(d)). Similarly, TBARs levels in liver homogenates and isolated mitochondria were not elevated in the TNF*α* plus pyrazole-treated CYP2E1 knockout mice but were increased in the wild-type mice. Normal liver pathology was observed after pyrazole plus TNF*α* treatment of CYP2E1 knockout mice. (Figure 2(e)). The failure of TNF*α* to induce liver injury in pyrazole-treated CYP2E1 knockout mice supports a critical role for CYP2E1 in the potentiated injury observed in the wild-type mice.

## 7. Mitochondrial Dysfunction

**Figure 3:**
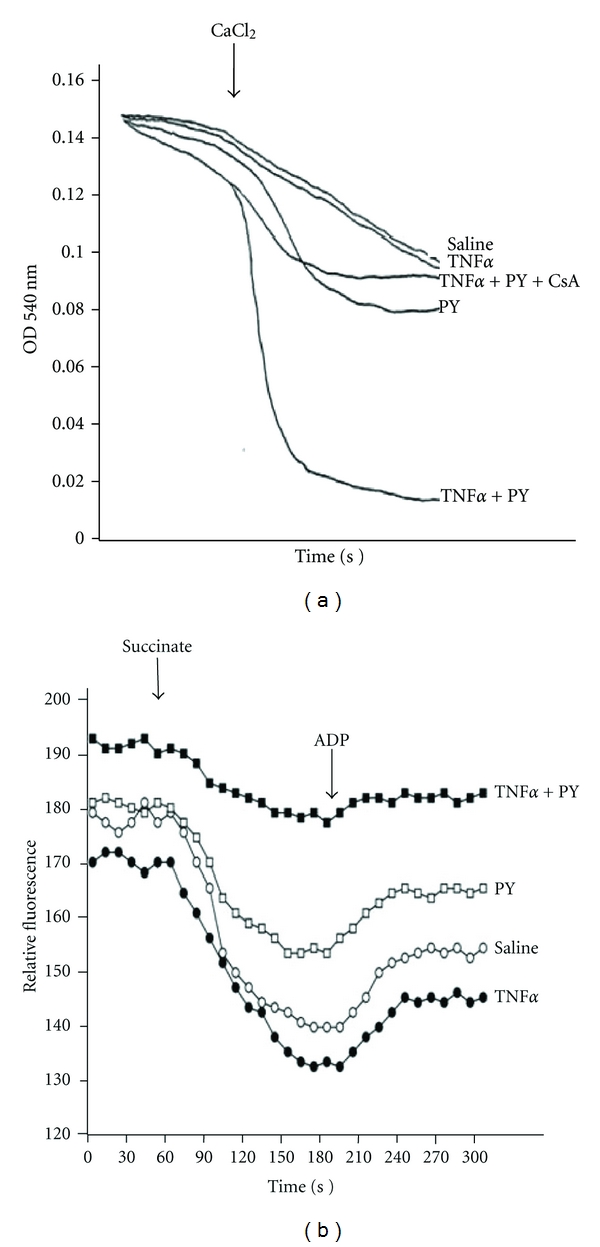
Treatment with TNF*α* plus pyrazole causes mitochondrial injury. TNF*α* plus pyrazole causes mitochondrial injury as reflected by (a) increased mitochondrial swelling (decreased absorbance at 540 nm) or (b) decreased mitochondrial membrane potential as assayed by succinate-dependent decline in rhodamine 123 fluorescence. The decline in fluorescence after addition of succinate is reflective of the mitochondrial membrane potential. Note: this decline is very small in the mitochondria isolated from TNF*α* plus pyrazole-treated mice. In (a) the increased swelling produced by TNF*α* plus pyrazole is prevented by cyclosporine A (CsA), an inhibitor of the mitochondrial permeability transition.

 Alcohol can cause mitochondrial dysfunction [75, 76]. We hypothesized that mitochondria are an eventual target for developing liver injury induced by TNF*α* when CYP2E1 is elevated by pyrazole. Initiation of a mitochondrial permeability transition was determined by assessing mitochondrial swelling in the absence and presence of 100 *μ*M calcium. Succinate (10 mM) was the respiratory substrate. As shown in Figure 3(a), in the absence of calcium, swelling (decrease in absorbance at 540 nm) was low with all mitochondrial preparations although there was some basal swelling with the mitochondria from the pyrazole plus TNF*α*-treated mice. The addition of 100 *μ*M calcium caused a low rate of swelling in the saline or TNF*α* alone mitochondria; swelling was somewhat elevated in the pyrazole alone mitochondria. Swelling was very rapid without any lag phase with the mitochondria from the pyrazole-plus-TNF*α*-treated mice (Figure 3(a)). Importantly, this rapid swelling was blocked by cyclosporine A (2 *μ*M), a classic inhibitor of the mitochondrial permeability transition. Calcium elevates mitochondrial swelling in the saline-, TNF*α* alone-, and pyrazole alone groups, which was most pronounced in the TNF*α* plus pyrazole group. The calcium-induced swelling was sensitive to cyclosporine A in all groups. The basal swelling, in the absence of added calcium, was also higher in the TNF*α* plus pyrazole group, further suggestive of mitochondrial dysfunction. 

 The electrochemical potential of the proton gradient generated across the mitochondrial membrane (ΔΨ) was assessed by monitoring fluorescence quenching of rhodamine 123. Addition of 10 mM succinate at one minute caused a decrease in fluorescence reflective of a high ΔΨ corresponding to state 4 of respiration (Figure 3(b)). The decline in fluorescence averaged about 40 arbitrary units per minute with mitochondria from the saline or TNF*α* alone treated mice and 30 arbitrary units per minute with mitochondria from the pyrazole-treated mice. However, the decline in fluorescence was only about 14 arbitrary units with mitochondria from the TNF*α*-plus-pyrazole-treated mice. Addition of ADP at 3 minutes caused an enhancement of fluorescence which corresponds to state 3 respiration as part of the proton motive force is utilized to synthesize ATP. This enhancement of fluorescence averaged 15, 14, 12, and 4 arbitrary units per minute for mitochondria from the saline, TNF*α* alone, pyrazole alone, and TNF*α* plus pyrazole treated mice, respectively. Taken as a whole, these initial data suggest a small decline in ΔΨ in mitochondria from the pyrazole-treated mice and a more pronounced decline in mitochondria from the pyrazole-plus-TNF*α*-treated mice.

## 8. Cyclosporine A (CsA) Prevents Pyrazole Plus LPS-Induced Liver Injury [77]

 We evaluated whether cyclosporine A (CsA), an inhibitor of the mitochondrial permeability transition, could protect against the TNF*α*-plus-pyrazole-induced liver injury. Such an experiment could validate that mitochondrial dysfunction is a key downstream target in this injury. Male C57BL/6 mice were treated with saline, pyrazole, LPS, or pyrazole plus LPS plus corn oil or pyrazole plus LPS plus 1 dose of CsA (100 mg/kg body wt, dissolved in corn oil). Serum ALT and AST levels were elevated in the PY + LPS + corn oil group compared to the other 3 groups. CsA treatment attenuated this increase in transaminases. H&E staining of liver tissue showed that the PY + LPS + corn oil treatment induced extensive liver zonal necrosis and that the CsA treatment prevented this. Mitochondrial swelling was increased in mitochondria isolated from the PY + LPS + corn oil treated mice compared to mitochondria from the saline + corn oil mice. The in vivo treatment with CsA prevented this increase in mitochondrial swelling, which likely explains the protection against LPS-plus-pyrazole-induced liver injury. The LPS plus pyrazole elevation of 4-HNE and 3-NT protein adducts were also decreased by CsA, suggesting that mitochondrial dysfunction plays an important role in the increase in oxidative/nitrosative stress.

## 9. Activation of MAP Kinases

 Mitogen-activated protein kinases (MAPKs) are serine-threonine kinases that mediate intracellular signaling associated with a variety of cellular activities including cell proliferation, differentiation, survival, death, and transformation. The mammalian MAPK family consists of extracellular signal-regulated kinase (ERK), p38 MAPK, and c-Jun NH2-terminal kinase (JNK; also known as stress-activated protein kinase or SAPK) [78]. The MAPK signaling cascade consists of three distinct members of the protein kinase family, including MAP kinase (MAPK), MAPK kinase (MAPKK), and MAPKK kinase (MAPKKK). MAPKKK phosphorylates and thereby activates MAPKK, and the activated form of MAPKK in turn phosphorylates and activates MAPK. Activated MAPK may translocate to the cell nucleus and regulate the activities of transcription factors and thereby control gene expression [79, 80]. In either in vivo or in vitro models of alcoholic liver disease, an increase of gene expression of the MAPK pathway was found [81, 82]. Compatible data in protein expression levels were seen in many studies. Intraperitoneal injection of alcohol to rats induced rapid phosphorylation of p38 MAPK, and JNK after only 1 hr of ethanol injection, and this was accompanied with apoptosis of the liver [83]. In human stellate cells, increased phosphorylation of p38 MAPK and JNK was found to be associated with ethanol-induced stellate cell activation, toxicity, and apoptosis [84]. JNK and p38 MAPK may become activated simultaneously, while some studies have shown that JNK and p38 MAPK may even react in the opposite way according to the specific treatments. In one study, after chronic alcohol feeding, LPS stimulation of Kupffer cells increased p38 MAPK activity, whereas it decreased JNK activity [85]. In human monocytes, acute alcohol exposure increased JNK phosphorylation, while chronic alcohol exposure decreased JNK activity [86]. Apparently, further studies are needed to clarify why MAPK can react differently depending on the stimuli or in different cell lines. 

**Figure 4:**
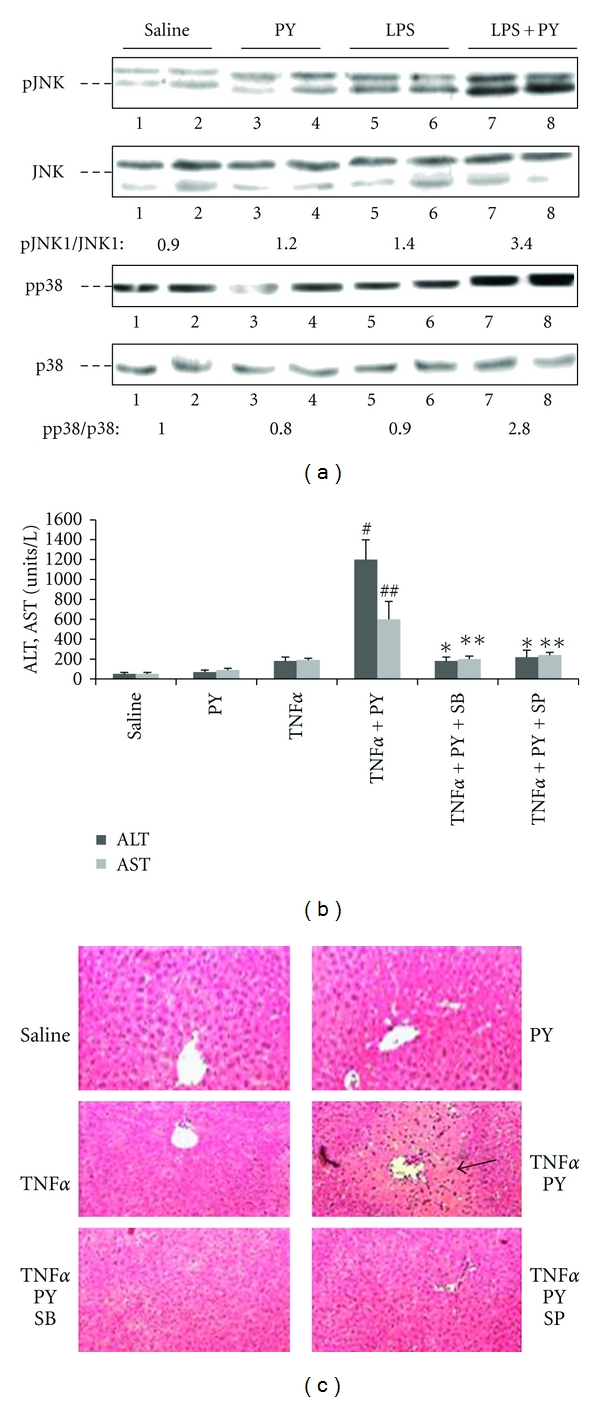
MAP kinase activation. (a) LPS plus pyrazole treatment activates JNK and p38 MAPK. The pJNK/JNK and the pp38 MAPK/p38MAPK ratios are shown below the blots. (b) Either the JNK inhibitor SP600125 (SP) or the p38 MAPK inhibitor SB203508 (SB) prevents TNF*α* plus pyrazole-induced elevation of ALT and AST or (c) liver pathology.

 MAP kinases such as JNK or p38 MAPK have been shown to play important roles in several models of liver injury, including CYP2E1-dependent toxicity [69, 70, 87–92]. We evaluated possible activation of MAP kinases in our pyrazole/LPS or pyrazole/TNF*α* hepatotoxicity models by assaying for the phosphorylated MAPK. As shown in Figure 4(a), LPS treatment alone did not cause significant JNK activation or p38 MAPK activation as reflected by the low p-JNK and pp38 MAPK levels relative to total JNK and p38 MAPK levels. Similar low ratios were found for the saline or the pyrazole alone treated mice (Figure 4(a)). However, both JNK and p38 MAPK were activated in livers of the pyrazole plus LPS-treated mice. A similar activation of JNK and p38 MAPK was observed after pyrazole plus TNF*α* but not in mice treated with TNF*α* or pyrazole alone [73]. ERK was not altered by TNF*α* alone or pyrazole plus TNF*α* treatment. To evaluate the significance of these changes in MAPK activation, the effect of SP600125, an inhibitor of JNK, and SB203580, an inhibitor of p38MAPK, on the hepatotoxicity was determined. The TNF*α* plus pyrazole elevation of transaminases was blunted by administration of SP600125 (15 mg/kg) or SB203580 (15 mg/kg) (Figure 4(b)). The MAPK inhibitors also lowered the necrosis (Figure 4(c)) and partially blocked the increased oxidative stress produced by the pyrazole plus TNF*α* treatment, but had no effect on CYP2E1 activity or protein levels. These results suggest the CYP2E1 elevation of TNF*α* liver injury and oxidative stress is MAPK dependent. The activation of JNK in the pyrazole plus TNF*α* group was blocked by SP600125 but not SB203580 whereas the activation of p38 MAPK was blocked by SB203580 but not SP600125.

## 10. Activation of ASK-1 and Downstream Map Kinase Kinases

**Figure 5:**
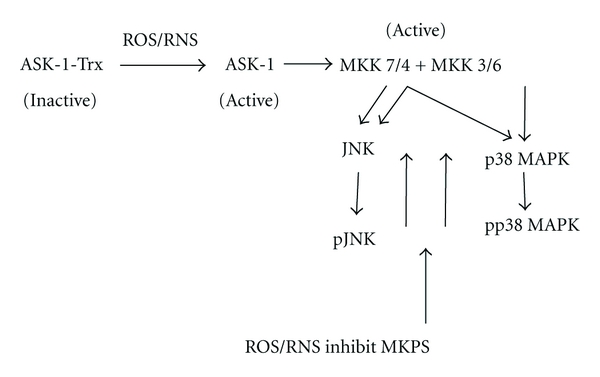
Scheme for activation of ASK-1 by ROS/RNS and downstream MAP kinase kinases (MKK4/7 and MKK 3/6) and MAP kinase JNK and p38 MAPK by ROS/RNS. Dissociation of the inhibitory thioredoxin (TRX) from the TRX-ASK-1 complex by ROS/RNS activates ASK-1. MKPS and MAP kinase phosphatases which deactivate activated JNK and p38 MAPK by dephosphorylation are inhibited by ROS/RNS thereby sustaining the activation of JNK and p38 MAPK.

 The upstream mediators of JNK and p38 MAPK activation were not identified in these previous studies. For mechanistic and therapeutic implications, it would be important to evaluate the MAP kinase kinase kinase and MAP kinase kinase which activate JNK and p38 MAPK in this PY plus TNF*α* model. Apoptosis signal-regulating kinase 1 (ASK-1) is a member of the MAP3K family which is responsive to stress-induced cell damage. Activation of ASK-1 can determine cell fate by regulation of both the MKK4/MKK7-JNK and the MKK3/MKK6-p38 MAPK signaling cascades [93]. ASK-1 is activated by oxidative stress, ER stress, and inflammatory cytokines such as TNF*α* [94]. In resting cells, ASK-1 forms an inactive complex with reduced thioredoxin (Trx). Under conditions of stress by TNF*α* or ROS, ASK-1 dissociates from Trx and becomes activated [95] (Figure 5). Oxidation of Trx by ROS causes dissociation of ASK-1 from the oxidized Trx which switches the inactive form of ASK-1 to the active kinase. The Trx-ASK complex is thought to be a redox sensor, which functions as a molecular switch turning the cellular redox state into a MAP kinase signaling pathway [96]. Activated ASK-1 then promotes activation (phosphorylation) of the downstream MAPKK, MKK4/MKK7 which can activate JNK, and MKK3/MKK6 which can activate p38 MAPK [93–96] (Figure 5). We evaluated whether CYP2E1 plus-TNF*α*-induced ROS promote release of ASK-1 from the Trx-ASK1 complex and activate ASK-1 followed by the phosphorylation of MKK4/MKK7 and/or MKK3/MKK6 which subsequently regulate the phosphorylation of JNK and p38 MAPK and contribute to the liver injury.

 Wild-type mice treated with PY plus TNF*α* developed liver injury between 8 and 12 h after TNF*α* administration as reflected by the high levels of ALT and AST at 12 h. Oxidative stress is a likely key factor to trigger signaling and liver injury in CYP2E1-mediated hepatotoxicity [97]. A time course for oxidative stress after PY plus TNF*α* treatment was studied. GSH was decreased in wild-type mice after 4 h and remained at lower levels for at least 12 h as compared to the TNF*α* alone group. Lipid peroxidation increased significantly at 4 h in the PY-plus-TNF*α*-treated mice and remained elevated up to 12 h. These results show that oxidative stress occurs at an earlier time after administration of TNF*α* than does liver injury in the TNF*α*-plus-PY-treated mice. Treatment with PY increased the levels of CYP2E1 prior to the administration of TNF*α*, and CYP2E1 levels remained about 2-fold elevated at least until 8 h after administration of TNF*α* in the PY-treated mice. 

 Since previous results showed a key role of JNK and p38 MAPK in the TNF*α*-plus-PY-induced liver injury, we evaluated whether upstream MAPKK and MAPKKK were activated, the time course for their activation in relation to the hepatic injury, and the role of CYP2E1. We focused on ASK-1 since this MAPKKK has been shown to be important as a target for TNF*α* signaling [93, 94, 96]. TNF*α* or pretreatment with PY alone did not activate ASK-1. TNF*α* plus PY treatment activated ASK-1 3-fold compared with the 0 hour control at 4 h after TNF*α* treatment. Activation of ASK-1 decreased at 8 and 12 h. Immunoprecipitation experiments showed that ASK-1 was bound to Trx-1 at 0 h but was released from the Trx-ASK1 complex at 4 h and remained free from binding to Trx1 at 8 and 12 h. No ASK-1 release from the Trx-ASK1 complex was found in TNF*α* alone treated mice. ASK-1 was not activated in PY-plus-TNF*α* treated CYP2E1−/− mice, and no ASK-1 was released from the Trx-ASK1 complex in CYP2E1−/− mice. Thus, activation of ASK-1 by treatment with TNF*α* plus PY is associated with its release from the Trx-ASK1 complex, occurs prior to the liver injury, and requires CYP2E1. 

MKK4/7 and MKK3/6 are the MAPKK which activate downstream JNK or p38 MAPK, respectively, [98]. They are also targets for activation by ASK-1 [99, 100]. Treatment of wild-type mice with PY plus TNF*α* activated MKK4 at 4, 8, and 12 h compared with the TNF*α* alone groups [101]. No activation of MKK4 was found in TNF*α* or TNF*α* + PY treated CYP2E1−/− mice [92]. MKK7 was activated only at 12 h. MKK3 was activated as early as 4 h in the TNF*α*-plus-PY-treated mice, while MKK6 was activated at 8 h. JNK was activated in the TNF*α* + PY mice at 8 and 12 h, and p38 MAPK was activated at 12 h when compared with TNF*α* alone. In CYP2E1−/− mice, neither MKK4/7, MKK3/6, JNK, nor p38 MAPK was activated. Thus, the time course experiments suggest MKK4 may be the MAPK responsible for activation of JNK, while either MKK3 or MKK6 may be the MAPKK responsible for the activation of p38 MAPK. 

 In summary, a time course of in vivo liver injury induced by PY plus TNF*α* was carried out to determine the sequence of events and relationships between induction of CYP2E1, oxidative stress, the activation of ASK-1, MKK3/MKK6, MKK4/MKK7, p38 MAPK and JNK with the development of liver injury [101]. The liver injury occurs at 8 to 12 h after the addition of TNF*α*. Since ROS is postulated to be a critical factor in the mechanism by which TNF*α* plus PY induce liver injury, development of ROS should precede the liver injury. Indeed, hepatic GSH levels were decreased and TBARS levels were elevated 4h after administration of TNF*α* to PY-treated mice. Thus oxidative stress precedes the liver injury. A likely contributor to the increase in oxidative stress is the induction of CYP2E1 by the pyrazole treatment as no injury or oxidative stress was observed in CYP2E1 knockout mice. CYP2E1 levels were already elevated at the time of TNF*α* administration (0 h) since the mice were treated for two days prior to this injection of TNF*α* on day 3. ASK-1, a member of the MAPKKK family, activates both MKK4/MKK7-JNK and MKK3/MKK6-p38 MAPK signaling cascades. ASK-1 was activated in PY-treated mice at 4 h after the administration of TNF*α*. Immunoprecipitation analysis showed that ASK-1 was dissociated from the inactive Trx-ASK complex at 4 h, consistent with the activation of ASK-1 at 4 h. In CYP2E1−/− mice, pyrazole plus TNF*α* treatment failed to activate ASK-1 and ASK-1 was not dissociated from the Trx-ASK1 complex. If CYP2E1-generated ROS is important for the release and activation of ASK-1, elevation of CYP2E1 and in oxidative stress should occur as early events. Increases in CYP2E1 and ROS occur at 4 h, at least consistent with the activation of ASK-1 at 4 h, although future experiments with shorter time intervals will be necessary to evaluate these relationships in more detail. Our results implicate a role for ASK-1 in CYP2E1 potentiation of TNF*α*-induced liver injury. Future experiments with ASK-1 knockout mice [102] would be interesting to further validate the role of ASK-1 in the PY/TNF*α* model. JNK or p38 MAPK activities are increased upon phosphorylation by MAPK kinase (MKK4/MKK7 or MKK3/MKK6) [98]. The activity of ASK-1 modulates and regulates the phosphorylation of MKK4/MKK7 and MKK3/MKK6. PY plus TNF*α* treatment increased MKK4 phosphorylation at 4, 8, and 12 h, while activation of MKK7 was delayed until 12 h. MKK3 and MKK6 phosphorylations were also increased at 4 to 8 h. In CYP2E1−/− mice, no MAPKK was activated at any observation time point. TNF*α* alone did not significantly activate the MAPKK in wild-type or CYP2E1−/− mice. The activation of MKK4 and MKK3/6 (4–8 h) occur prior to the onset of liver injury (8–12 h). 

 The role of CYP2E1 in the activation of ASK-1, MKK4/MKK7 or MKK3/MKK6 is apparent, since TNF*α* treatment only induced such activations in wild type mice treated with PY to induce CYP2E1 but not in CYP2E1−/− mice. We hypothesize that TNF*α* alone- or CYP2E1 alone-generated ROS stress is not sufficient to trigger the dissociation of ASK-1 from the Trx-ASK complex. The CYP2E1 sensitization of TNF*α*-induced liver injury may occur through a synergistic effect with TNF*α* to produce an enhanced ROS stress consistent with the so-called “Two Hit” hypothesis. We speculate that similar interactions between CYP2E1 and TNF*α* may be important for alcohol-induced liver injury.

## 11. Thioredoxin-CYP2E1-ASK-1-JNK1 Interactions

 The thioredoxin system plays a key role in modulating redox signaling pathways which regulate physiological as well as pathophysiological processes [103, 104]. The thioredoxin system includes thioredoxin, thioredoxin reductase, and thioredoxin peroxidases. Thioredoxin has a conserved catalytic site (-Cys-Gly-Pro-Cys-Lys-) that undergoes reversible oxidation to the cystine disulfide. Oxidized thioredoxin is a major substrate for thioredoxin reductase, and reduced thioredoxin serves as an electron carrier to reduce peroxiredoxins. The oxidized thioredoxin is reduced back to the reduced form by thioredoxin reductase [105, 106]. There are two main thioredoxins: thioredoxin-1 (TRX-1), a cytosolic form; thioredoxin-2 (TRX-2), a mitochondrial form [105]. Modification of thiols in thioredoxin interrupts signaling mechanisms involved in cell growth, proliferation, and apoptosis. The role of thioredoxin in the regulation of the activation of apoptosis signal-regulating kinase-1 (ASK-1) and downstream apoptosis pathways has been reported in multiple studies [95, 96, 106, 107]. Thioredoxin can associate with the N-terminal portion of ASK-1 in vitro and in vivo. Expression of thioredoxin inhibited ASK-1 kinase activity and the subsequent ASK-1-dependent apoptosis [107]. In resting cells, endogenous ASK-1 constitutively forms a complex which includes thioredoxin. Upon ROS stimulation, the ASK-1 unbinds from thioredoxin and forms a fully activated higher-molecular-mass complex. As discussed above, TNF*α* increases oxidative stress in mice with elevated CYP2E1, with subsequent activation of ASK-1 via a mechanism involving thioredoxin-ASK-1 dissociation, followed by activation of downstream MKK and MAPK [101]. 

 Both TRX-1 and TRX-2 are involved in the protection from oxidative stress. TRX-2 plays an important role in protecting the mitochondria against oxidative stress and in protecting cells from ROS-induced apoptosis. Supplementation of human recombinant TRX-1 to mice fed a Lieber-DeCarli ethanol diet decreased several markers of oxidative stress, inflammatory cytokine expression, and apoptosis in liver [108]. Since thioredoxin is a reducing molecule which can decrease oxidative stress, we evaluated [109] whether thioredoxin can inhibit the oxidative stress induced by CYP2E1, and whether there is any difference in the function of TRX-1 versus TRX-2 in blunting CYP2E1 oxidant stress. SiRNA for either TRX-1 or TRX-2 was added to HepG2 cells with CYP2E1 expression (E47 cells) or without CYP2E1 expression (C34 cells) to test (1) whether thioredoxin decreases oxidative stress and injury induced by CYP2E1; (2) considering the compartmentation of thioredoxin, whether TRX-1 or TRX-2 has a stronger protective effect in preventing against this injury and oxidative stress; (3) what the mechanism of the protection by thioredoxin from cell death in CYP2E1-expressing cells is [109].

 Both E47 and C34 cells were treated with either control siRNA, TRX-1 siRNA, or TRX-2 siRNA, or both TRX-1 and TRX-2 siRNA for 72 hrs. TRX-1 expression was decreased by 90% by either TRX-1 siRNA alone or TRX-1 and TRX-2 siRNA together in both cell lines. TRX-2 expression was decreased by 80–90% by TRX-2 siRNA alone or TRX-1 and TRX-2 siRNA together in both cell lines. TRX-1 siRNA is specific for cytosolic thioredoxin and had no effect on levels of mitochondrial thioredoxin, and TRX-2 siRNA is specific for decreasing mitochondrial thioredoxin and had no effect on levels of cytosolic thioredoxin. 

**Figure 6:**
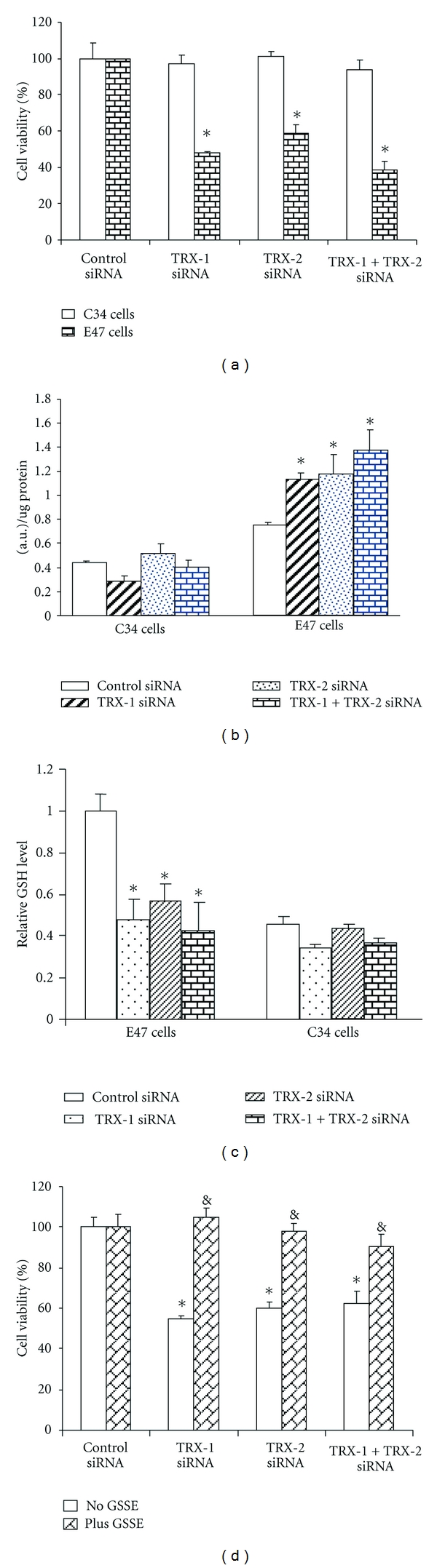
Effect of thioredoxin (TRX) knockdown on E47 (express CYP2E1) and C34 (do not express CYP2E1) HepG2 cell viability. E47 and C34 cells were treated with control siRNA or cytosolic TRX-1 siRNA or mitochondrial TRX-2 siRNA or both TRX-1 and TRX-2 siRNAs for 72 hours. (a) Cell viability was determined by a MTT assay. (b) ROS production was determined by a fluorescence assay. Arbitrary units of fluorescence by the E47 and C34 cells. (c) Cellular levels of glutathione (GSH). The GSH level in each group was expressed as the value relative to that of the control siRNA treatment group in E47 cells. (d) Supplementation with GSH restores E47 cell viability after TRX knockdown. At 24 hours, 5 mM glutathione ethyl ester (GSSE) was added to the cell culture medium, and the cells were incubated with the indicated siRNA for 48 hours followed by MTT assay. Note: both cytosolic and mitochondrial TRX are important in protection of HepG2 cells from CYP2E1-generated oxidant stress.

 Knockdown of TRX-1 or TRX-2 or both decreased cell viability of E47 cells by 40–60%, but cell viability of C34 cells was not affected with the knockdown of either TRX-1 or TRX-2 or both (Figure 6(a)). These results indicate that cell death induced by thioredoxin knockdown under these conditions is CYP2E1 dependent and that decreasing either TRX-1 or TRX-2 promotes this toxicity. To assess the mode of cell death, experiments studying uptake of propidium iodide or annexin V staining were carried out. Uptake of propidium iodide into E47 cells was elevated upon knockdown of either TRX-1 or TRX-2 or both. Annexin V staining, taken as a reflection of apoptosis, was also elevated in the E47 cells upon knockdown of TRX-1 or TRX-2 or both. Thus, the cell death appears to be a mix of necrosis plus apoptosis, that is, necroptosis. We next evaluated whether knocking down of thioredoxin intracellularly by siRNA induces ROS production and lipid peroxidation. Total ROS was detected both by fluorescence microscopy, flow cytometry assay, and spectrofluorimetry assay. An increase of ROS production was detected in E47 cells but not in C34 cells after 72 hrs treatment with either TRX-1 or TRX-2 siRNA or both. Quantification of ROS production by spectrofluorimetry indicated that total ROS production was elevated 50–100% by thioredoxin knockdown in E47 cells (Figure 6(b)). There were no increases in ROS production in C34 cells upon thioredoxin knockdown. There were significant increases of ROS production when either TRX-1 or TRX-2 was lowered. This suggests that TRX-1 alone or TRX-2 alone is not sufficient to protect the E47 cells from oxidative stress. It would appear that both TRX-1 or TRX-2 are essential for the protection of E47 cells from oxidative stress. The production of superoxide was assayed using dihydroethidium (DHE) as the probe. Knockdown of TRX-1 or TRX-2 or both increased DHE fluorescence in E47 cells, but not C34 cells. 4-HNE adduct formation was analyzed by immunocytochemistry with fixed E47 and C34 cells. At baseline, 4-HNE adduct expression is higher in E47 cells than C34 cells when control siRNA was applied, similar to the increase in fluorescence of E47 compared to C34 cells. There was no increase of 4-HNE adducts in C34 cells, but a significant increase of 4-HNE adducts was observed in E47 cells when comparing either TRX-1 or TRX-2 or both siRNA treatment to control siRNA treatment. Treatment with either TRX-1 or TRX-2 siRNA or both did not cause a significant change of total glutathione level in C34 cells, while a 50% decrease was found in E47 cells (Figure 6(c)). This suggests that with knockdown of thioredoxin, glutathione was consumed as a major reducing molecule and antioxidant. Addition to the culture medium of glutathione ethyl ester prevented E47 cell death caused by either TRX-1 or TRX-2 siRNA or both together (Figure 6(d)). The lowering of, as well as the protection by, glutathione suggests that the knockdown of thioredoxin-induced cell death is related to oxidative stress. 

**Figure 7:**
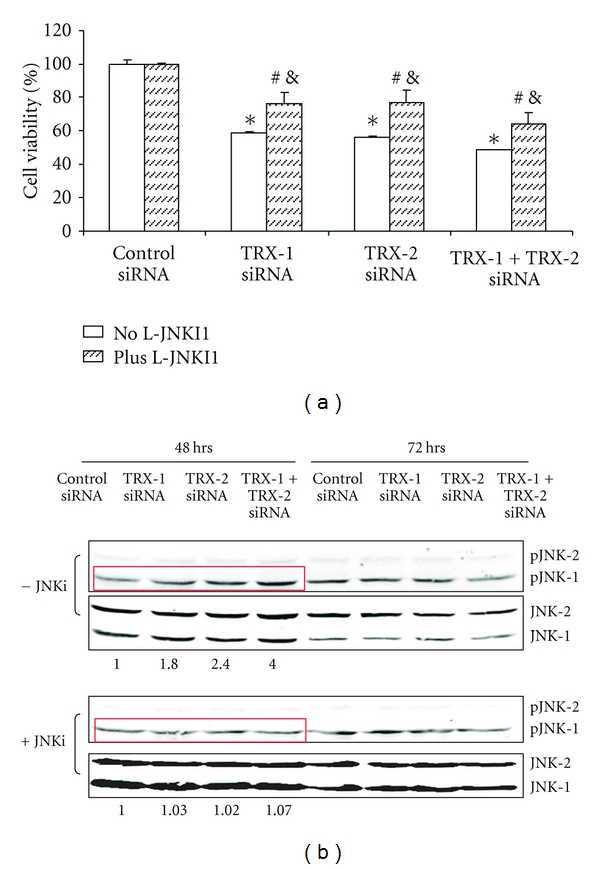
A JNK inhibitor protects the E47 cells from loss of viability produced by TRX knockdown. The E 47 cells were incubated with and without 5 uM of the JNK inhibitor L-JNKI1for 3 hours followed by treatment with the indicated siRNA for 48 or 72 hours. (a) MTT assay to determine cell viability. (b) The effect of TRX knockdown on the activation of JNK in the absence and presence of the JNK inhibitor. Numbers under the blots refer to the pJNK/JNK ratio.

 Since thioredoxin is bound to ASK-1 and inhibits the activation of ASK-1, experiments were carried out to evaluate whether thioredoxin knockdown activates ASK-1 and downstream MAPK signaling pathways in the E47 cells. Increased ASK-1 phosphorylation was seen by immunohistochemistry in E47 cells upon treatment with TRX-1 or TRX-2 siRNA or both at 5, 24, and 48 hrs, but not after 72 hrs of siRNA treatment. Western blot analysis revealed a 2–4-fold increase in the pASK-1/ASK-1 ratio 24 hrs and 48 hrs but not after 72 hrs of thioredoxin knockdown. ASK-1 activates downstream MAPK such as JNK and p38 MAPK, ultimately by promoting their phosphorylation to pJNK or pp38 MAPK [95, 96]. Increased JNK1 but not JNK2 phosphorylation was seen in E47 cells treated with either TRX-1 or TRX-2 siRNA for 48 hrs (Figure 7(b)). No such activation persisted at 72 hrs after treatment. Thus, activation of JNK1 occurs after the earlier activation of ASK-1 (5–48 hrs) and declines when activation of ASK-1 terminates (72 hrs). p38 MAPK was not activated under these conditions as there was no increase in pp38 MAPK levels. One downstream target of JNK1 is cJUN phosphorylation. There was an increase in the pc-JUN/c-JUN ratio 72 hrs after treatment with siRNA for TRX-1, TRX-2, or both, a time point after the activation of JNK1 (48 hrs). 

Could the CYP2E1 plus thioredoxin knockdown induced cell death be mediated through ASK-1 and JNK1 signaling pathways? The JNK inhibitor, L-JNKI1, which specifically inhibits the phosphorylation of JNK, lowered the decline in E47 cell viability from 45–50% in the absence of L-JNKI1 to about 20–30% in the presence of L-JNKI1 plus TRX-1, or TRX-2 siRNA, or both TRX-1 and TRX-2 siRNA treatment (Figure 7(a)). Under these conditions, L-JNKI1 strongly blunted the activation of JNK which occurs 48 hrs after thioredoxin knockdown; the pJNK1/JNK1 ratio was elevated 2- to 4-fold by siRNA for TRX-1 or TRX-2 or both in the absence of JNKI1, whereas no increase in pJNK1/JNK1 was observed in the presence of the inhibitor (Figure 7(b)). The partial protection by L-JNKI1 suggests that the cell death induced by thioredoxin knockdown was partly via JNK signaling pathways, although non-JNK-dependent pathways are also likely involved.

 In conclusion, both cytosolic and mitochondrial thioredoxin are important in protecting HepG2 cells from cell death by oxidative stress induced by CYP2E1. Thioredoxin knockdown increased cellular production of ROS and increased lipid peroxidation in HepG2 cells expressing CYP2E1. The signaling pathway which induced cell death by thioredoxin knockdown may involve, at least in part, the activation of ASK-1 and JNK1. This protection by both TRX-1 and TRX-2 against CYP2E1-dependent toxicity may play a role in the ability of thioredoxin to protect against ethanol-induced hepatotoxicity [108] and suggests that antioxidative protection in both the cytosol and mitochondria is necessary for effective protection against liver injury potentiated by CYP2E1.

## 12. Effect of N-Acetylcysteine (NAC)

 We evaluated [74] the effect of NAC, a general antioxidant and a precursor of GSH, on the potentiation of TNF*α* toxicity by pyrazole as a proof of principle that oxidative stress plays an important role in the overall liver injury. C57BL/6 mice were treated with pyrazole for two days and then challenged with either saline or TNF*α*. Some mice in each group were also treated with 150 mg/kg NAC on the second day of treatment with pyrazole and on day 3 prior to the challenge with TNF*α*. The elevation in ALT and AST and the necrosis caused by the pyrazole plus TNF*α* treatment were lowered by NAC. The increase in TBARs produced by pyrazole plus TNF*α* and the decline in liver GSH were both prevented by NAC. Treatment with NAC had no effect on CYP2E1 protein levels or CYP2E1 catalytic activity. The activation of JNK or p38 MAPK by the pyrazole plus TNF*α* treatment, compared to pyrazole alone, was blocked by NAC. The pyrazole plus TNF*α* treatment elevated levels of iNOS 2.6-fold, and this increase in iNOS was blunted by NAC to a 1.4-fold increase. These results with NAC suggest that elevated oxidative stress is central to the activation of JNK and p38 MAPK, to peroxynitrite formation, and to the liver injury produced by treatment with pyrazole plus TNF*α*.

## 13. Pyrazole/TNF***α*** Hepatotoxicity in iNOS Knockout Mice

**Figure 8:**
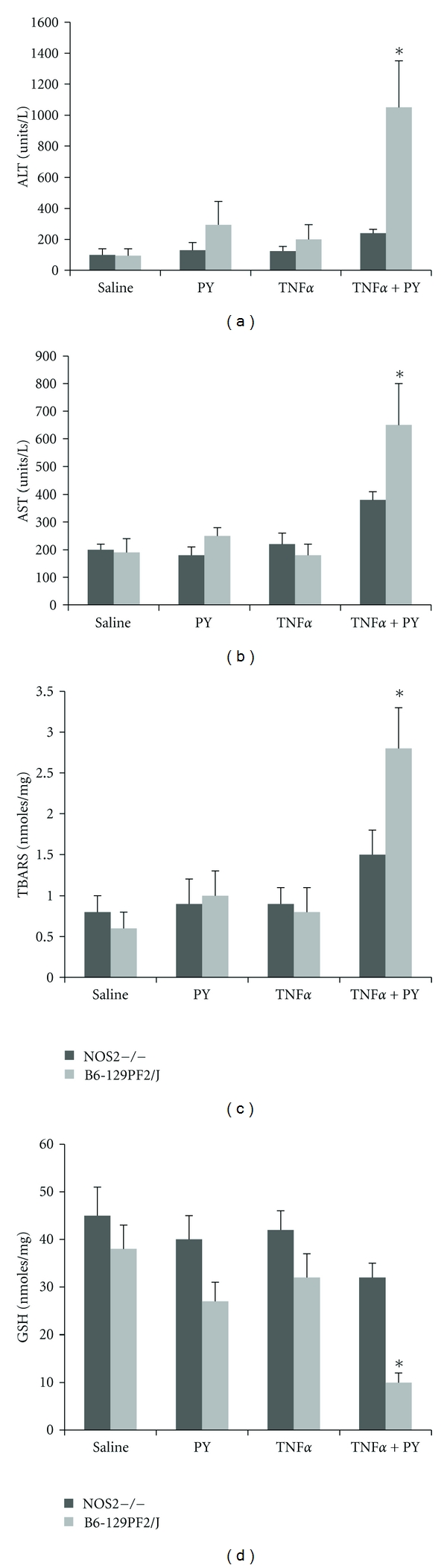
TNF*α*-plus-pyrazole-induced hepatotoxicity and oxidative stress are decreased in iNOS knockout mice. B6-129 WT mice and B6-129 iNOS knockout mice (NOS2−/−) were treated with either saline or pyrazole alone or TNF*α* alone or pyrazole plus TNF*α* for 3 days followed by assays of (a) ALT/AST, (b) TBARS, and (c) GSH. Note: liver injury and oxidant stress were much lower in the NOS2−/− mice than the WT mice indicating a role for NO and NO metabolites in the TNF*α*-plus-pyrazole-induced liver injury and oxidative stress.

The inducible nitric oxide synthase (iNOS) has been shown to play an important role in alcohol-induced liver injury [110]. We hypothesized that induction of CYP2E1 by pyrazole and induction of iNOS by LPS/TNF*α* result in the formation of the powerful oxidant peroxynitrite, ONOO, derived from the reaction between O_2_^−•^ and NO. 3-Nitrotyrosine protein adducts (3-NT) were elevated in the liver after pyrazole plus LPS treatment [71, 72]. We believe that ONOO plays a key role in the oxidative/nitrosative stress and hepatotoxicity produced by the pyrazole plus LPS/TNF*α* treatment. If correct, oxidative/nitrosative stress and hepatotoxicity produced by pyrazole plus LPS/TNF*α* treatment should be blunted in iNOS null mice. NOS2 (iNOS) knockout mice (B6-129P2) and genetic background control B6-129PF2/J mice were purchased from Jackson Laboratory and treated with saline or pyrazole alone or TNF*α* alone or pyrazole plus TNF*α*. The pyrazole plus TNF*α* treatment elevated ALT or AST levels about 2-fold (*P* < 0.05) in iNOS null mice as compared to treatment with saline or pyrazole alone or TNF*α* alone (Figure 8(a)). Pyrazole plus TNF*α* elevated ALT and AST about four- to fivefold in the genetic background mice (Figure 8(a)) (*P* < .01 compared to the increase in ALT and AST in iNOS null mice). In NOS2−/− mice, TNF*α* plus PY induced some hepatocyte degeneration change in the pericentral area but no loci of necrosis were found. In the control wild-type B6-129PF2/J mice, TNF*α* plus PY induced more severe liver injury and necrotic loci were found in several pericentral areas. TNF*α* plus PY slightly increased lipid peroxidation in NOS2−/− mice compared with saline-, PY-, or TNF*α*-treated mice. Lipid peroxidation was more significantly elevated by TNF*α*-plus-PY treatment in B6-129PF2/J mice (4-fold increase) compared to the other groups and to the TNF*α* plus PY treated NOS2−/− mice (2-fold increase, Figure 8(b)). TNF*α* plus PY lowered GSH levels by 25% in NOS2−/− mice, while a more pronounced decline in GSH occurred in the control mice (67% decrease, Figure 8(c)). Levels of CYP2E1 were elevated to comparable extents by pyrazole in the wild-type and the iNOS knockout mice (about 2.5–3-fold); thus, the lower liver injury in the iNOS knockout mice is not due to lower levels of CYP2E1. These results suggest that while TNF*α* plus PY does induce some liver injury and oxidant stress in the NOS2−/− mice, a more severe liver injury and oxidant stress is induced by TNF*α* plus PY in the control mice. We hypothesize that NO derived from iNOS reacts with superoxide radical produced from CYP2E1 to generate the powerful oxidant peroxynitrite which plays a critical role in the liver injury produced by TNF plus pyrazole. The absence of iNOS in the knockout mice with the accompanying decline in NO would prevent formation of significant amounts of peroxynitrite even though superoxide continues to be produced from the elevated CYP2E1 and therefore liver injury is lowered.

## 14. Conclusions

**Figure 9:**
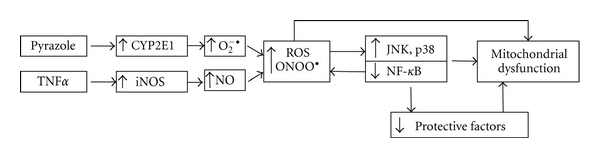
Model for the potentiation of TNF*α*-induced hepatotoxicity, oxidative stress mitochondrial dysfunction, and activation of MAPK by pyrazole induction of CYP2E1. Pyrazole induction of CYP2E1 coupled to TNF*α* induction of iNOS results in elevated oxidative/nitrosative stress in hepatocytes. This results in activation of JNK and p38 MAPK which, along with the elevated ROS/RNS, damage mitochondrial function ultimately leading to liver injury.

 This paper has focused on two major contributors to mechanisms by which ethanol causes liver injury, induction of CYP2E1, and elevated endotoxin (LPS) levels followed by increased production of TNF*α*. Each of these has been extensively studied, but there are few studies in which both factors have been evaluated simultaneously. We have shown that induction of CYP2E1 by pyrazole potentiates LPS- or TNF-induced hepatotoxicity. Evidence for a role for CYP2E1 comes from studies in which the CYP2E1 inhibitor CMZ blocks the liver injury, and from studies with CYP2E1 knockout mice where pyrazole plus LPS toxicity is blunted. The potentiated toxicity is associated with an increase in oxidative and nitrosative stress. Prevention of such increases, for example, treatment with the antioxidant NAC or administration of TNF*α* plus pyrazole to iNOS knockout mice, blunts the liver injury thus validating that the elevated oxidative/nitrosative stress plays a key role in producing the liver injury rather than occurs because of liver injury. JNK and P38 MAP kinases are activated by the combined pyrazole plus LPS/TNF*α* treatment. Preventing activation of JNK with SP600125 or activation of P38 MAPK with SB203580 decreases the liver injury. Inhibition of CYP2E1 or use of CYP2E1 knockout or iNOS knockout mice or preventing the oxidative/nitrosative stress decreases the activation of JNK and P38 MAPK. We hypothesize that the increase in oxidative/nitrosative stress and the activation of MAP kinases ultimately impact on mitochondrial integrity and function as shown by the increase in mitochondrial swelling and decline in mitochondrial membrane potential. Protection of mitochondrial integrity with cyclosporine A prevents the TNF*α*-plus-pyrazole-induced hepatotoxicity and oxidative stress. In HepG2 cells expressing CYP2E1, both cytosolic and mitochondrial TRX are necessary for protection against CYP2E1-generated oxidative stress, and cell toxicity. We hypothesize that similar interactions involving activation of MAP kinases, oxidative stress and mitochondrial dysfunction occur as a result of ethanol induction of CYP2E1 and elevation of LPS/TNF*α*, and our working scheme is shown in Figure 9. Induction of CYP2E1 by pyrazole or ethanol increases superoxide radical production, while elevation of LPS/TNF*α* by ethanol activates iNOS and NO production. The interaction between superoxide and NO produces the powerful oxidant peroxynitrite. Downstream targets for ROS and RNS include activation of ASK-1 and subsequently JNK and mitochondrial dysfunction which contribute to loss of viability.
